# A general framework for extrapolation-aware prediction reliability in forward and inverse analyses of Gaussian mixture regression models

**DOI:** 10.1007/s44211-026-00924-y

**Published:** 2026-06-01

**Authors:** Hiromasa Kaneko

**Affiliations:** Department of Applied Chemistry, School of Science and Technology, Meiji University, 1-1-1 Higashi-Mita, Tama-ku, Kawasaki, Kanagawa 214-8571 Japan

**Keywords:** Gaussian mixture regression, Direct inverse analysis, Prediction reliability, Applicability domain, Probability density

## Abstract

Gaussian mixture regression (GMR) and direct inverse analysis (DIA) are powerful tools for molecular, material, and process design, but their reliability decreases when extrapolation occurs beyond the training data. To address this challenge, an index of extrapolation (IoE) that evaluates extrapolation potential and prediction trustworthiness in both forward and inverse analyses of GMR models is proposed. Defined as the negative logarithm of the probability density function, the IoE enables stable identification of interpolation-like and extrapolation-like regions. Validation with numerical simulations confirmed its ability to distinguish these regions, while applications to data sets of organic solubility, inorganic superconductivity, and batch processes demonstrated that high-IoE regions correspond to reduced prediction reliability, where prediction errors may show larger dispersion. The proposed IoE provides a practical and generalizable framework to assess applicability domains, enhancing the interpretability and reliability of machine learning models. This advancement strengthens data-driven design and supports more efficient discovery of novel molecules, materials, and processes.

**Graphical abstract:**

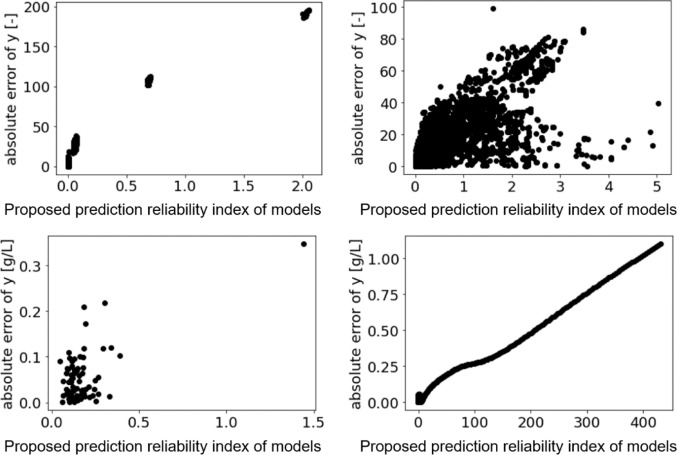

## Introduction

In molecular design, material design, process design, and process management, a mathematical model y = f(x) is constructed between features x such as molecular descriptors, experimental conditions, synthesis conditions, manufacturing conditions, evaluation conditions, process conditions, and process variables, and objective variables y such as physical properties, activities, and characteristics of molecules, materials, and product. y values are predicted by inputting x values into the model, which is forward analysis of the model, or x values for which y has target values are designed by inverse analysis of the model.

Inverse analysis of a model is merely a pseudo-inverse analysis that generates a large number of virtual samples of x, inputs them into the model to predict y values, and selects samples with good predicted y values, i.e., a vast repetition of forward analysis of the model. Optimization methods can be used to improve the efficiency of the forward analysis, but y is predicted only in the ranges of x set by humans, and x candidates cannot be optimized beyond the existing y. In addition, because the solution space in x increases exponentially with the number of x, it is impossible to perform an exhaustive forward analysis of the model, considering all the various conditions such as experimental, synthesis, manufacturing, evaluation, and process conditions. Although Bayesian optimization [1, 2], which has been the focus of attention in design of experiments and active learning, has made it possible to search for extrapolated regions of x using not only predicted y values but also their variance, Bayesian optimization is also a pseudo-inverse analysis of Gaussian regression models, and true inverse analysis that directly predicts causes (x) from results (y) is not possible.

A method was developed that directly predicts x values from y values, i.e., a true inverse analysis of a mathematical model [3]. This is called the direct inverse analysis (DIA) of the model. In the DIA method, after converting the experimental data of compounds, materials, and products into x, such as experimental, synthesis, manufacturing, evaluation, and process conditions, the relationship between x and y as a mathematical model is expressed as a joint probability density function by superposition of multiple Gaussian distributions, and from the multiplication theorem of probability and Bayes’ theorem, the posterior probability density function of x can be calculated for a given value of y. This function allows direct prediction of x values with high probability from target y values [4]. Various material designs have been achieved by the DIA method. For example, DIA using experimental data of thermoelectric conversion materials has successfully proposed innovative and reasonable experimental conditions (e.g., mixing ratio of each element and synthesis temperature) for thermoelectric conversion materials in which thermal conductivity, electrical conductivity, and Seebeck coefficient all exceed those of existing materials [5].

Although DIA of a model can directly predict x values by inputting y values into the model, the reliability of x values predicted with DIA of the model is not discussed. All machine learning and artificial intelligence models are data-dependent in their applicability domains, and the more input data is extrapolated, the less reliable the prediction. Due to the characteristics of the mixture of Gaussian distributions, extrapolation regions for x and y have a Gaussian distribution with weights concentrated at a single point. However, this concentration can also occur in interpolation regions. Therefore, the presence of a single point of concentration does not necessarily mean that it is an extrapolation for x and y. In addition, the variance–covariance matrix of x obtained by inputting y values is a fixed value, and thus, the variance does not increase as much as extrapolation.

This study focuses on the probability density function (PDF) of Gaussian mixture models (GMM) and proposes an index to evaluate the reliability of the predictions of forward and inverse analysis of Gaussian mixture regression (GMR) models. Based on the joint probability distribution of x and y in GMM, the conditional probability distribution of y given x as GMR is calculated when predicting y from x, and the conditional probability distribution of x given y as GMR when predicting x from y, and then, in both cases, the PDF is employed. A small PDF value indicates that no existing samples exist in near neighbor.

The PDF value indicates the “relative data density at that point,” but the absolute value itself has no probabilistic meaning. Therefore, using the “PDF value itself” as a threshold to determine the extrapolation region is mathematically unstable because it depends too heavily on the scale of variables. Because the PDF value can become extremely small, a logarithmic transformation is applied to the PDF, and then, the result is multiplied by negative to obtain the index of extrapolation (IoE). A larger IoE value indicates greater extrapolation potential.

In this study, extrapolation is considered not only as a purely geometric separation in the numerical descriptor space but also as domain-specific chemical extrapolation. In molecular and material design, extrapolation often means predicting compounds or materials containing atom types, functional groups, elemental species, or elemental combinations that are absent from or insufficiently represented in the training data. This chemical extrapolation is particularly important in practical design because the goal is often to discover novel molecules or materials outside known chemical domains. Such chemically extrapolated samples are expected to be located in low-density regions of the descriptor or composition space after descriptor calculation, variable selection, and autoscaling. The proposed IoE evaluates this decrease in probability density through the GMR/GMM framework.

The effectiveness of the proposed method is verified using numerical simulation data, and its practicality is confirmed by applying the method to an organic compound data set, an inorganic compound data set, and time series data of batch processes.

## Proposed method

GMR is a regression analysis approach based on the use of GMMs. In this study, the GMM calculation uses mixture.GaussianMixture [6] and the variational Bayesian GMM calculation uses mixture.BayesianGaussianMixture [7] from the scikit-learn library; the GMR calculations use DCEKit [8]. The model determined is referred to as “GMR model” [9].

Although GMR model can predict x values directly by inputting y values into the model, and vice versa, not all predicted x and y values can be trusted. When the input y or x values are an extrapolation of an existing data domain, the predictions are likely to be unreliable, however the reliability of the predictions has not been discussed. In this study, an index IoE to quantitatively evaluate the reliability of GMR predictions is proposed.

For example in predicting x from y, the posterior probability distribution *p*(**x**|**y**) of x for a given **y** is given as follows:

1 $$p\left( {{\mathbf{x}}|{\mathbf{y}}} \right) = \sum\limits_{i = 1}^{n} {w_{{{\mathrm{y}},i}} p\left( {{\mathbf{x}}|{\mathbf{y}},{{\boldsymbol{\upmu}}}_{{{\mathrm{y}},i}} ,{{\boldsymbol{\Sigma}}}_{{{\mathrm{yy}},i}} } \right)} ,$$

where

2 $$w_{{{\mathrm{y}},i}} = \frac{{\pi_{i} p\left( {{\mathbf{y}}|{{\boldsymbol{\upmu}}}_{{{\mathrm{y}},i}} ,{{\boldsymbol{\Sigma}}}_{{{\mathrm{yy}},i}} } \right)}}{{\sum\limits_{j = 1}^{n} {\pi_{j} p\left( {{\mathbf{y}}|{{\boldsymbol{\upmu}}}_{{{\mathrm{y}},j}} ,{{\boldsymbol{\Sigma}}}_{{{\mathrm{yy}},j}} } \right)} }}.$$

In the extrapolation regions for x and y, the weight of the Gaussian distribution in Eq. (2) is concentrated at a single point due to the characteristics of the mixed Gaussian distribution, that is, the weight of one Gaussian distribution in Eq. (2) is one and the weights of the other Gaussian distributions is zero. However, the weight can be concentrated at a single point even in interpolation regions, and a single point of concentration does not necessarily mean that it is an extrapolation of x and y. The reason why a single point of concentration in extrapolation and a single point of concentration in interpolation are handled in the same way is because they are normalized by the denominator in Eq. (2). In interpolation regions, only the probability density of y in one Gaussian distribution *i*, *p*(**y**|**μ**_y,*i*_, **Σ**_yy,*i*_), is large, and a single point of concentration occurs when *p*(**y**|**μ**_y,*i*_, **Σ**_yy,*i*_) in other Gaussian distributions is small, while in the extrapolation domain, *p*(**y**|**μ**_y,*i*_, **Σ**_yy,*i*_) of all the Gaussian distributions are small, and a Gaussian distribution with relatively large values of *p*(**y**|**μ**_y,*i*_, **Σ**_yy,*i*_) are normalized by the denominator in Eq. (2), resulting in the large value or one. Therefore, when predicting x values from y values, the value of *p*(**y**|**μ**_y,*i*_, **Σ**_yy,*i*_) in the Gaussian distribution *i* in Eq. (2) is focused on, and when predicting y values from x values, the value of *p*(**x**|**μ**_x,*i*_, **Σ**_xx,*i*_) is focused on, which is probability of density function (PDF) in each Gaussian distribution. The larger this value is, the more likely it is to follow a Gaussian distribution obtained from an existing data set; conversely, the smaller the value, the more likely it is to be extrapolated.

When predicting x values from y values, the following value of PDF is calculated, considering the weight for each Gaussian distribution:

3 $$\sum\limits_{i = 1}^{n} {\pi_{i} p\left( {{\mathbf{y}}|{{\boldsymbol{\upmu}}}_{{{\mathrm{y}},i}} ,{{\boldsymbol{\Sigma}}}_{{{\mathrm{yy}},i}} } \right)} .$$

The scale of the value of *p*(**y**|**μ**_y,*i*_, **Σ**_yy,*i*_) depends on y, x and samples. In addition, the values can be very small, and therefore, logarithmic transformation is conducted, and the following IoE is calculated:

4 $$IoE = - \ln \left( {\sum\limits_{i = 1}^{n} {\pi_{i} p\left( {{\mathbf{y}}|{{\boldsymbol{\upmu}}}_{{{\mathrm{y}},i}} ,{{\boldsymbol{\Sigma}}}_{{{\mathrm{yy}},i}} } \right)} } \right).$$

The smaller the value of IoE, the more the interpolation, and the larger the value of IoE, the more the extrapolation.

In the forward use of the GMR model, which predicts y from x, IoE is calculated for the input x and used as a diagnostic index of extrapolation. A large IoE indicates that the input x is located in a low-density region of the training data and that the prediction reliability may be reduced. Here, reduced reliability means that the uncertainty, or variance, of the prediction-error distribution is expected to be larger; it does not mean that the realized prediction error must be large for every individual sample. For example, even if prediction errors are assumed to follow a normal distribution, a larger variance increases the probability of large errors but can still yield a small realized error when the error is close to the center of the distribution. Therefore, IoE should be used as a qualitative reliability indicator for identifying predictions that should be interpreted with caution, rather than as a deterministic estimator of the exact prediction error.

In the inverse use of the GMR model, which predicts x from y, GMR-based DIA calculates the conditional distribution of x for a given target y and directly obtains a representative predicted x vector. In this computational procedure, one predicted x is obtained for each target y. The extrapolation degree of the target y is evaluated using IoE in the y space. When the target y is located outside or in a low-density region of the y distribution observed in the training data, the resulting inverse prediction should be regarded as extrapolative and interpreted with caution.

The proposed IoE can be regarded as a GMR-consistent density-based applicability-domain measure. General applicability-domain measures, such as Mahalanobis distance and k-nearest-neighbor distance, are also useful for evaluating separation from training samples. However, these measures are not derived from the conditional probability structure of GMR and do not directly incorporate the multimodal Gaussian components, mixture weights, and covariance matrices used in GMR/DIA. GMM log-likelihood is conceptually related to IoE because both are based on probability density; however, IoE is formulated specifically for evaluating the input side of GMR predictions in both forward and inverse analyses. Since no established extrapolation index specific to GMR has been reported, direct comparison with a GMR-specific baseline is not available. A systematic comparison with general applicability-domain measures will be an important topic for future work.

## Results and discussion

**Fig. 1:**
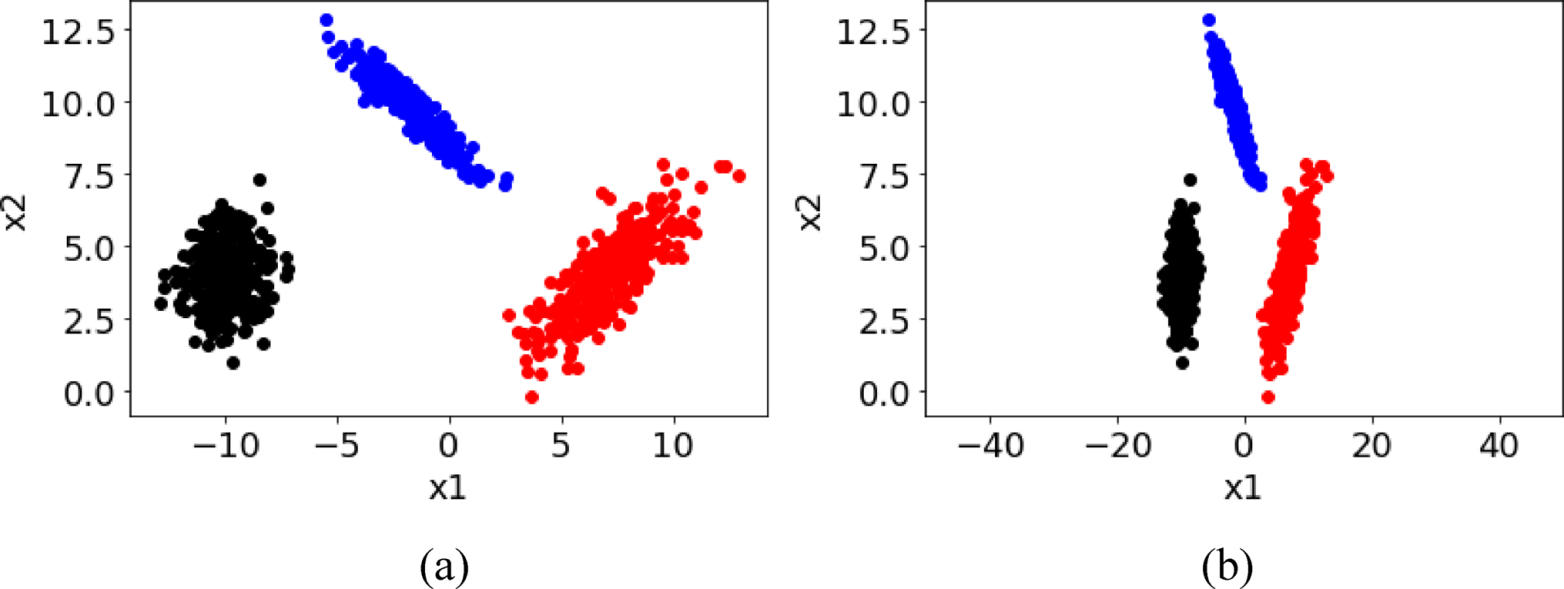
Numerical simulation data used. Differences in color signify differences in Gaussian distributions. **a** original, **b** extrapolation regions of × 1 are shown

To verify the effectiveness of the proposed IoE, numerical simulation data was used first. For two variables × 1 and × 2, three cluster of samples were generated based on Gaussian distributions as shown in Fig. 1. Each cluster contained 300 samples.

**Fig. 2:**
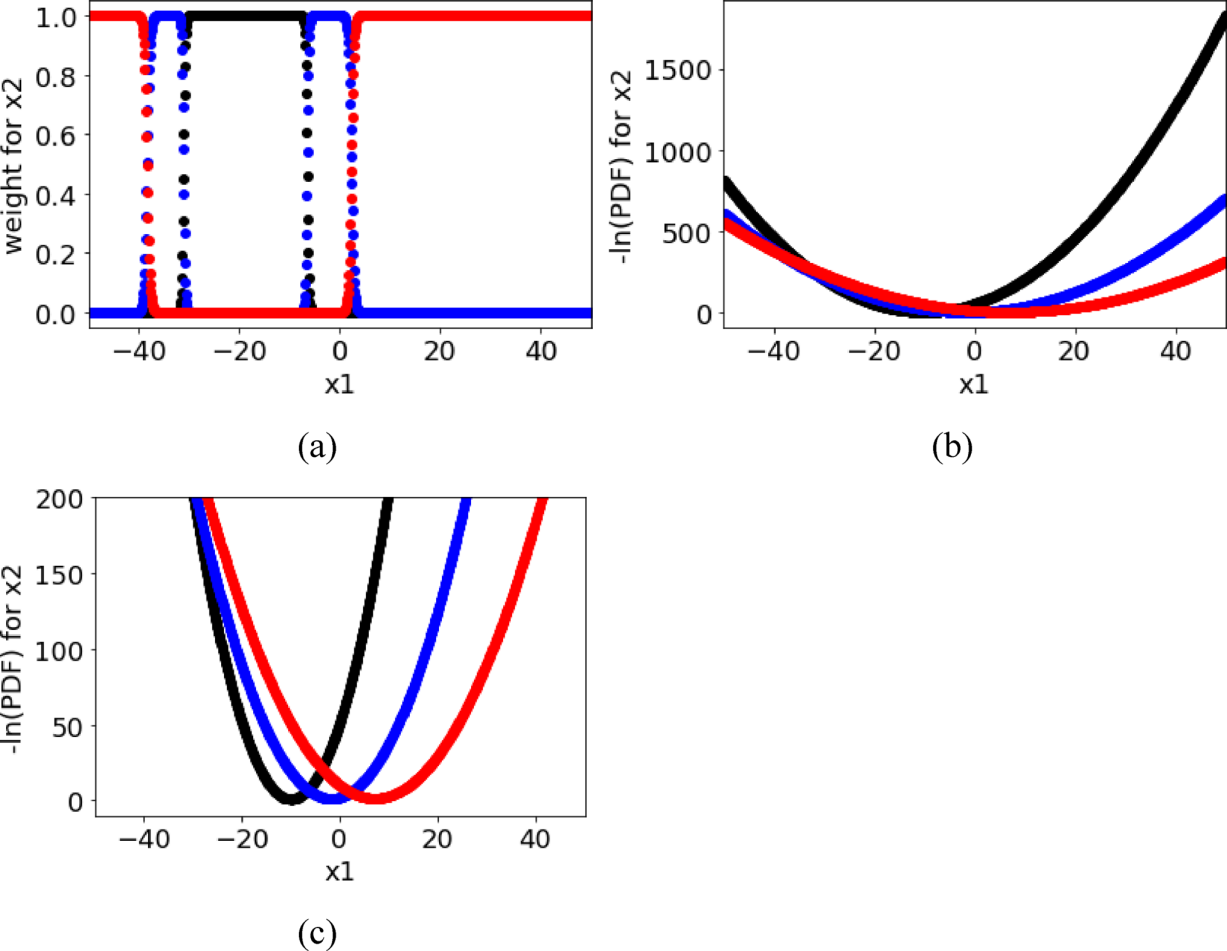
Weight and − ln(PDF) in Eq. (4) of each Gaussian distribution for × 2 when inputting × 1 values including the extrapolation regions. The colors correspond to those of the Gaussian distributions in Fig. 1. **a** weight, **b** − ln(PDF), **c** − ln(PDF) enlarged

A GMR model was constructed with the data set. Then, × 1 values from − 50 to 50, including the extrapolation regions of × 1 shown in Fig. 1b, were input into the GMR model, and the weights and PDF of each Gaussian distribution were output. Figure 2 shows the weight, and − ln(PDF) for each cluster before summation of Eq. (3). While the weight for one of the clusters became one in the extrapolation regions (× 1 ≤  − 15 or × 1 ≥ 15), the weight became one also in the interpolation region (− 15 ≤  × 1 ≤ 15). It was confirmed that the weights were not suitable as an index of extrapolation regions.

As shown in Fig. 2b and c, comparing with Fig. 1b, for samples with × 1 values within the range of each cluster, − ln(PDF) was small, indicating interpolation regions of × 1. Conversely, as × 1 values move away from the clusters, − ln(PDF) increased, confirming extrapolation regions of × 1. PDF could be used as an index of extrapolation regions.

**Fig. 3:**
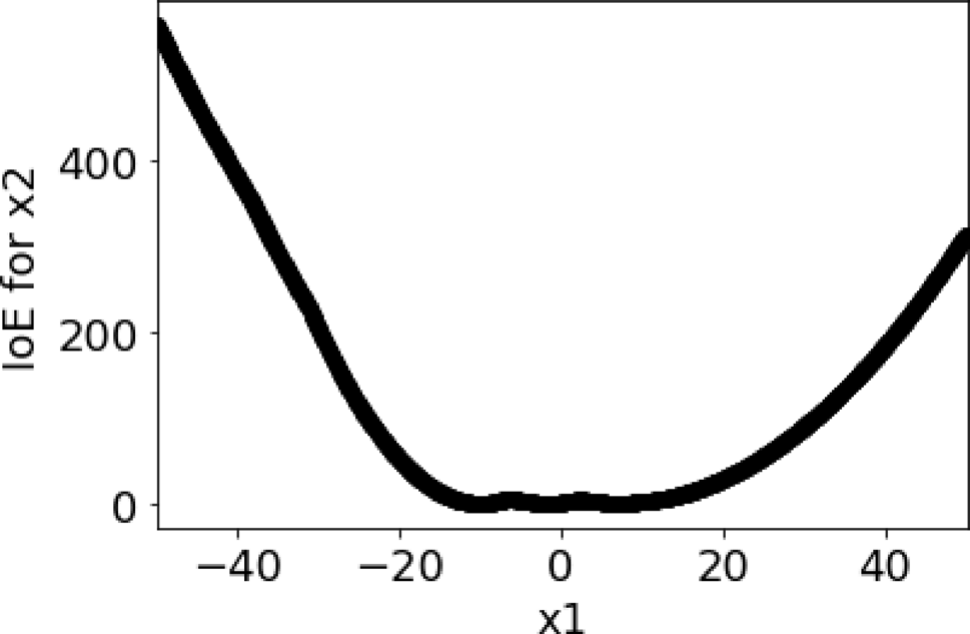
IoE for × 2 when inputting × 1 values including the extrapolation regions

Figure 3 shows the proposed IoE for × 2, calculated using the weights and PDF with Eqs. (3) and (4). Comparing with Fig. 1b, it was confirmed that IoE was small in the interpolation region (− 15 ≤  × 1 ≤ 15), indicating interpolation region of × 1. Conversely, IoE was large in the extrapolation regions (× 1 ≤  − 15 or × 1 ≥ 15), indicating extrapolation regions of × 1. The proposed IoE could quantitatively evaluate the extrapolation regions.

Then, IoE was applied to data sets of organic compounds, inorganic materials, and batch process simulations. The first data set of solubility in water (logS, S = solubility at 20–25 °C in moles per liter) [10] included 1290 organic compounds, and the second data set of superconducting (Tc) [11, 12] included 21,263 inorganic materials. For the logS data set, x consisted of two-dimensional molecular descriptors calculated with RDKit [13], and y was logS. RDKit afforded basic descriptors such as the number of atoms for each atom type and molecular weight, as well as descriptors including information on fragments, topology, and physicochemical properties. For the Tc data set, x consisted of the fractions of metal elements, and y was the logarithmically transformed critical temperature.

Before GMR modeling, no samples were removed as outliers. Variables in which 80% or more of the samples had the same value were removed because they contained little discriminatory information. For variable pairs with an absolute correlation coefficient of 0.9 or higher, one variable was removed to reduce severe multicollinearity. The remaining variables were autoscaled before modeling.

**Fig. 4:**
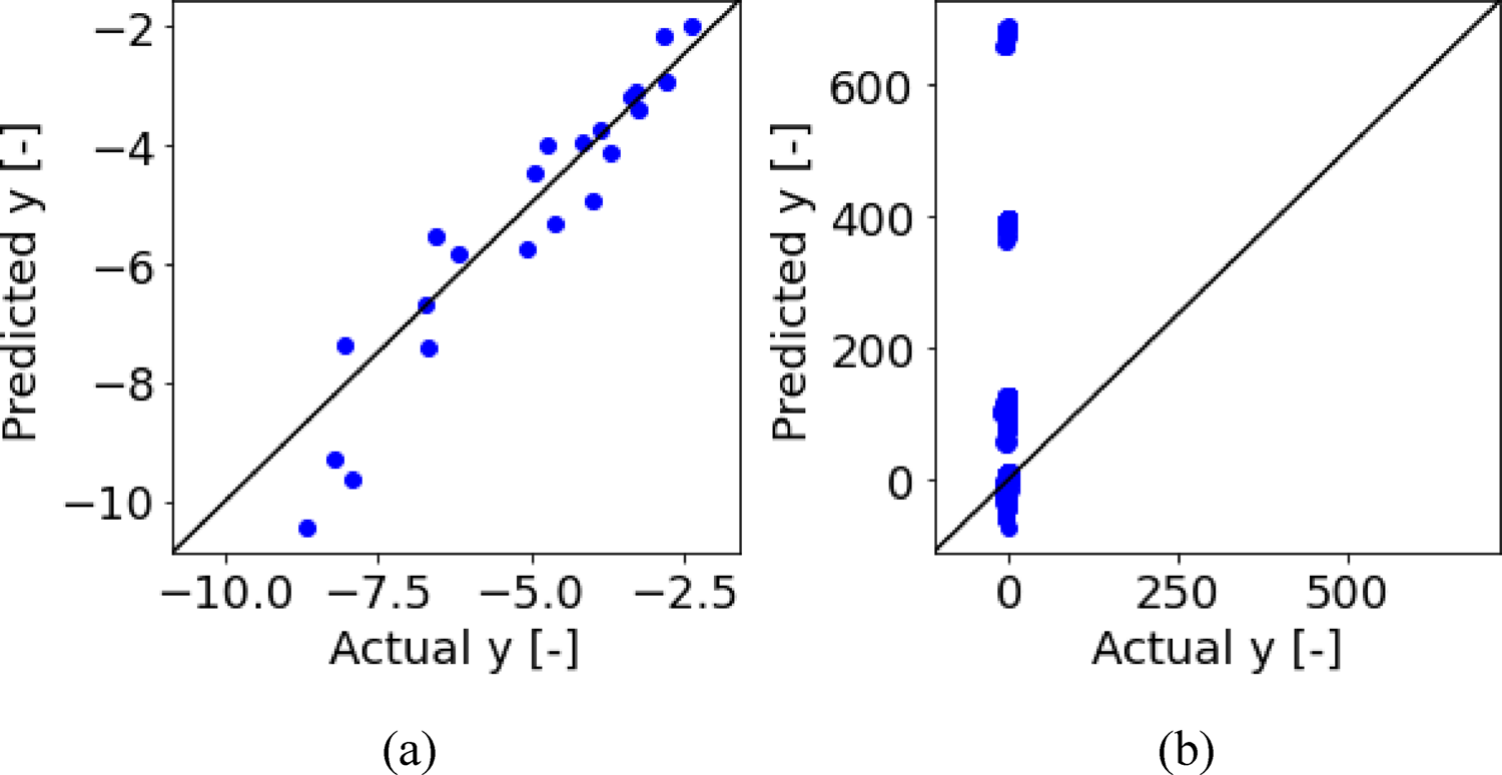
Actual y versus predicted y in test data of the logS data set. **a** Only hydrocarbon compounds, **b** all compounds

To clearly separate interpolation and chemical extrapolation in the logS data set, only hydrocarbon compounds were used to define the interpolation domain. Eighty percent of the hydrocarbon compounds were randomly selected as training data, while the remaining 20% hydrocarbon compounds were used as interpolation test data. Compounds other than hydrocarbons were used as chemically extrapolated test samples because they contain atom types, functional groups, or bonding environments that are not represented by the hydrocarbon-only training data. A GMR model was constructed using the training data, and the results of predicting y for the test data are shown in Fig. 4. From Fig. 4a, y was predicted well for hydrocarbon compounds, whereas prediction errors were larger for many compounds other than hydrocarbons, as shown in Fig. 4b.

**Fig. 5:**
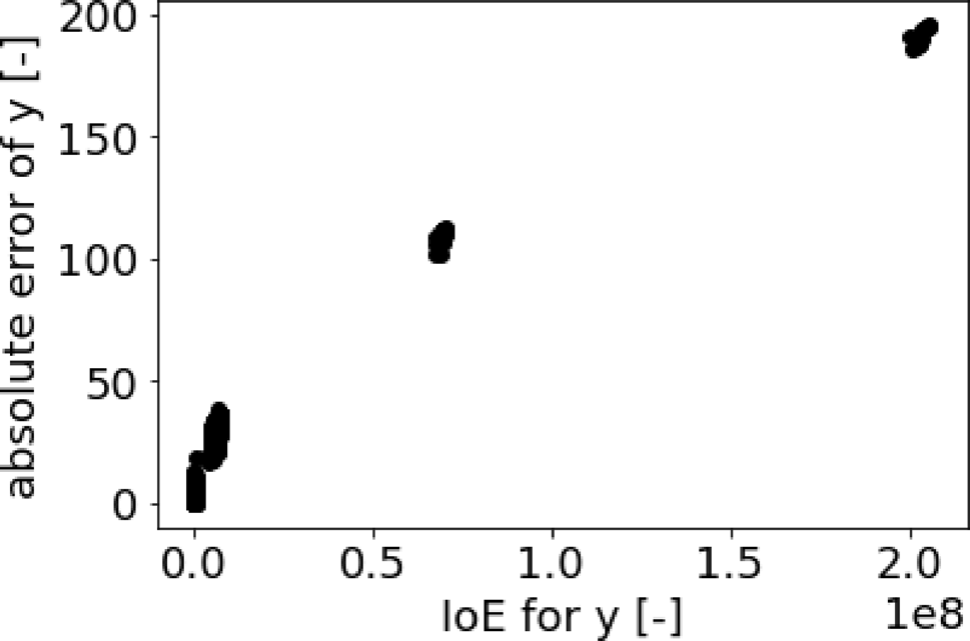
IoE for y versus absolute error of y in test data of the logS data set

Figure 5 shows the plot between IoE for y and the absolute prediction errors of y in the test data. High-IoE samples are located in low-density regions of the training data, indicating reduced prediction reliability. This reduced reliability should be interpreted as a larger expected dispersion of the prediction-error distribution. Therefore, a large IoE does not necessarily imply a large prediction error for every individual compound; even under a normal-error assumption, a prediction-error distribution with a large variance can still produce a realized error close to zero. These results confirm that IoE is useful for qualitatively evaluating extrapolation and prediction reliability in the GMR model.

**Fig. 6:**
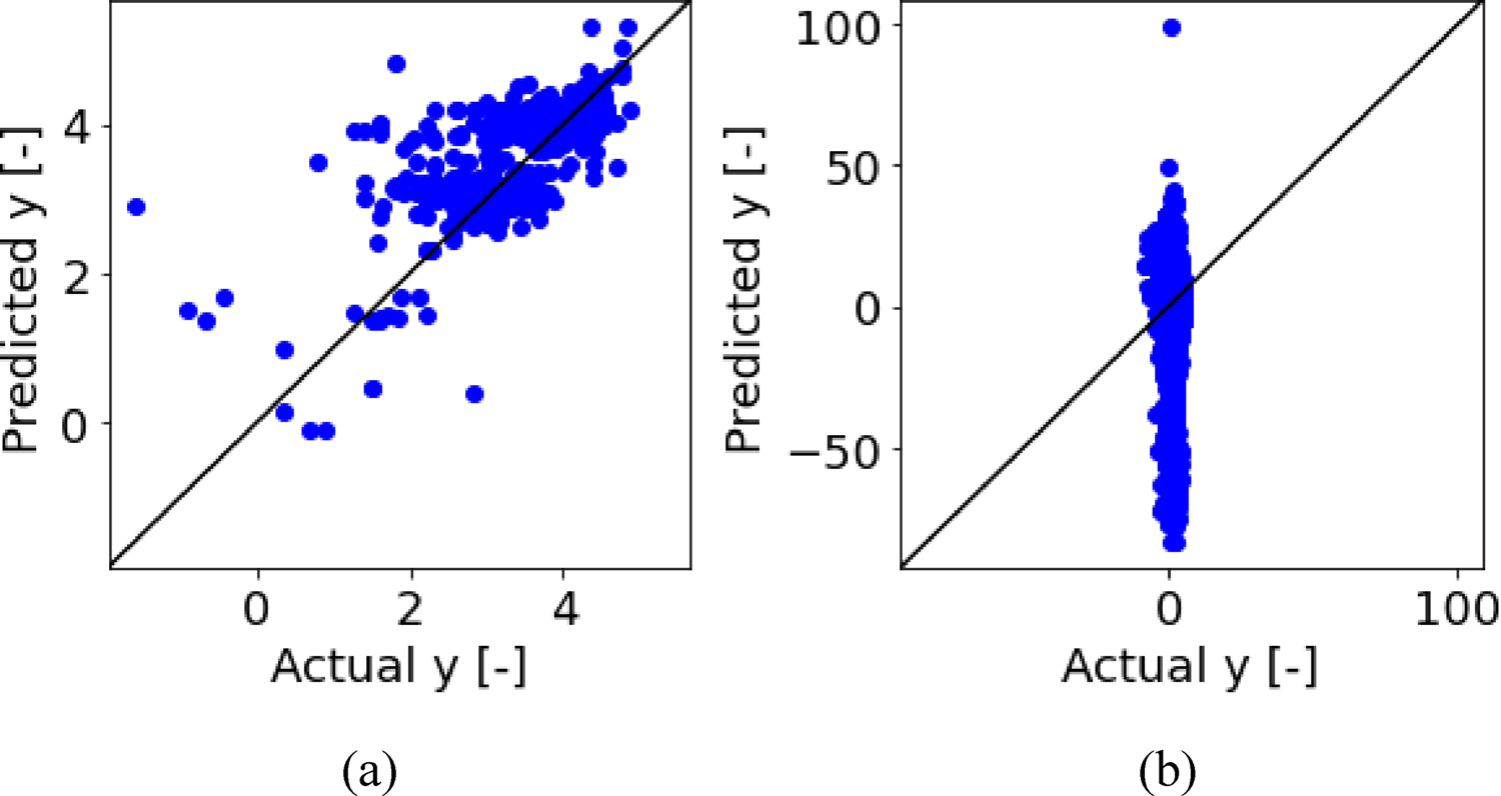
Actual y versus predicted y in test data of the Tc data set. **a** Only materials with the 13 elements, **b** all materials

To clearly separate interpolation and chemical/material extrapolation in the Tc data set, materials consisting only of the 13 most frequently appearing elements were used to define the interpolation domain. Eighty percent of these materials were randomly selected as training data, and the remaining 20% were used as interpolation test data. Materials containing elements other than the 13 elements were used as chemically extrapolated test samples because they include elemental species outside the training composition domain. Figure 6 shows the results of constructing a GMR model using the training data and predicting y for the test data. The y values were well predicted for materials composed only of the 13 elements, as shown in Fig. 6a, whereas prediction errors for many other materials were larger, as shown in Fig. 6b.

**Fig. 7:**
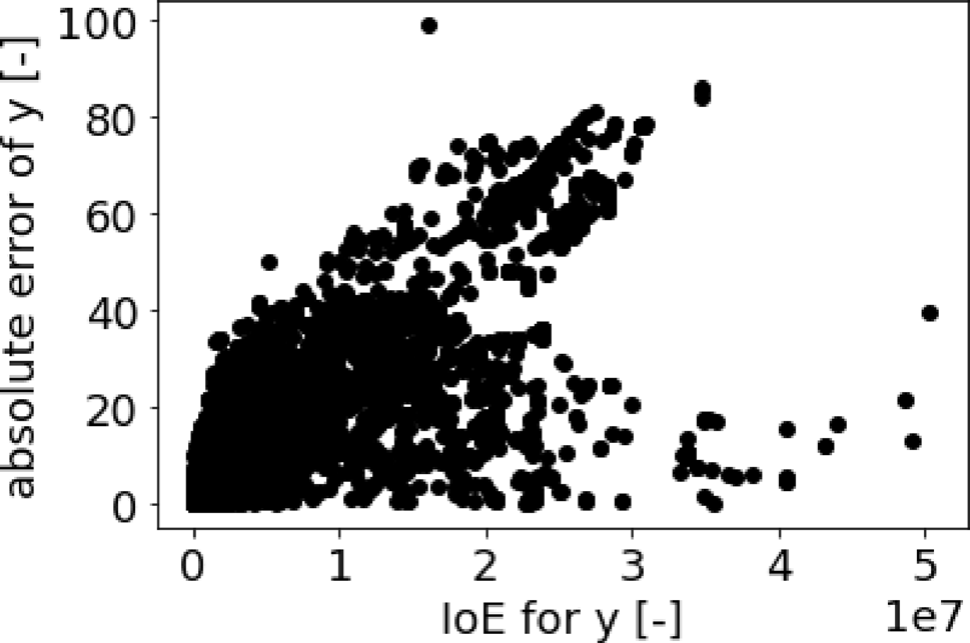
IoE for y versus absolute error of y in test data of the Tc data set

A plot between IoE for y and the absolute prediction errors of y for each material is shown in Fig. 7. High-IoE materials are located in low-density regions of the training data, and their predictions should be regarded as having reduced reliability. This means that the prediction-error distribution is expected to have larger dispersion in high-IoE regions, although an individual material can still have a small prediction error. As in the logS data set, IoE should be interpreted as a qualitative reliability indicator rather than an exact estimator of prediction error.

The proposed IoE was applied to a fed-batch bioreactor process [14, 15], wherein the mass balances are given as follows:

5 $$\frac{{d\left( {X\left( t \right)V\left( t \right)} \right)}}{dt} = V\left( t \right)r_{{\mathrm{g}}} \left( {X\left( t \right),S\left( t \right)} \right)$$

6 $$\frac{{d\left( {P\left( t \right)V\left( t \right)} \right)}}{dt} = V\left( t \right)r_{{\mathrm{p}}} \left( {X\left( t \right),S\left( t \right)} \right)$$

7 $$\frac{{d\left( {S\left( t \right)V\left( t \right)} \right)}}{dt} = F\left( t \right)S_{{\mathrm{f}}} \left( t \right) - \frac{1}{{Y_{X/S} }}V\left( t \right)r_{{\mathrm{g}}} \left( {X\left( t \right),S\left( t \right)} \right)$$

where *X*(*t*) is the cell concentration (g/L), *P*(*t*) is the product concentration (g/L), and *S*(*t*) is the substrate concentration (g/L). The unit of time is hours (h). The reactor is fed with fresh substrate at a concentration of *S*_f_(*t*) and a flowrate of *F*(*t*) (L/h). The volume *V*(*t*) (L) therefore changes according to the following relationship:

8 $$\frac{dV\left( t \right)}{{dt}} = F\left( t \right)$$

In addition, the rate *r*_g_(*X*(*t*), *S*(*t*)) represents the production of fresh cell biomass in units g/L/h. The cell specific growth is therefore expressed as follows:

9 $$r_{{\mathrm{g}}} \left( {X\left( t \right),S\left( t \right)} \right) = \mu \left( {S\left( t \right)} \right)X\left( t \right)$$

where *μ*(*S*(*t*)) is the cell specific growth rate. In the Monod model, the specific growth rate is a function of substrate concentration as follows:

10 $$\mu \left( {S\left( t \right)} \right) = \mu_{\max } \frac{S\left( t \right)}{{K_{{\mathrm{S}}} + S\left( t \right)}}$$

where *μ*_max_ is the maximum specific growth rate, and *K*_S_ is the half saturation constant, i.e., the value of *S*(*t*) for which *μ*(*S*(*t*)) = *μ*_max_/2. The product is assumed to be a by-product of cell growth as follows:

11 $$r_{{\mathrm{p}}} \left( {X\left( t \right),S\left( t \right)} \right) = Y_{P/X} r_{{\mathrm{g}}} \left( {X\left( t \right),S\left( t \right)} \right)$$

where *Y*_*P*/*X*_ is the product yield coefficient, which in turn is defined as follows:

12 $$Y_{P/X} = \frac{{{\mathrm{mass}}\,{\mathrm{of}}\,{\mathrm{product}}\,{\mathrm{formed}}}}{{{\mathrm{mass}}\,{\mathrm{of}}\,{\mathrm{new}}\,{\mathrm{cells}}\,{\mathrm{formed}}}}$$

The model further assumes that the amount of substrate consumed is proportional to the mass of new cells formed, where *Y*_*X*/*S*_ is the yield coefficient for new cells:

13 $$Y_{X/S} = \frac{{{\mathrm{mass}}\,{\mathrm{of}}\,{\mathrm{new}}\,{\mathrm{cells}}\,{\mathrm{formed}}}}{{{\mathrm{mass}}\,{\mathrm{of}}\,{\mathrm{substrate}}\,{\mathrm{consumed}}}}$$

where x is the time-series data of *F*(*t*) and *S*_f_(*t*), and y is *P*(*t*) at the endpoint. The number of batches was set to 300, the discretization interval was set at 0.5 [h], the batch time was randomly generated between 25 and 50 h, and *F*(*t*) and *S*_f_(*t*) were generated by random walks up to each batch time. Subsequently, the fed-batch reactor was simulated according to Eqs. (5)–(13) and y was computed. A total of 225 batches were randomly selected and used as training data, while the other 75 batches were used as test data.

**Fig. 8:**
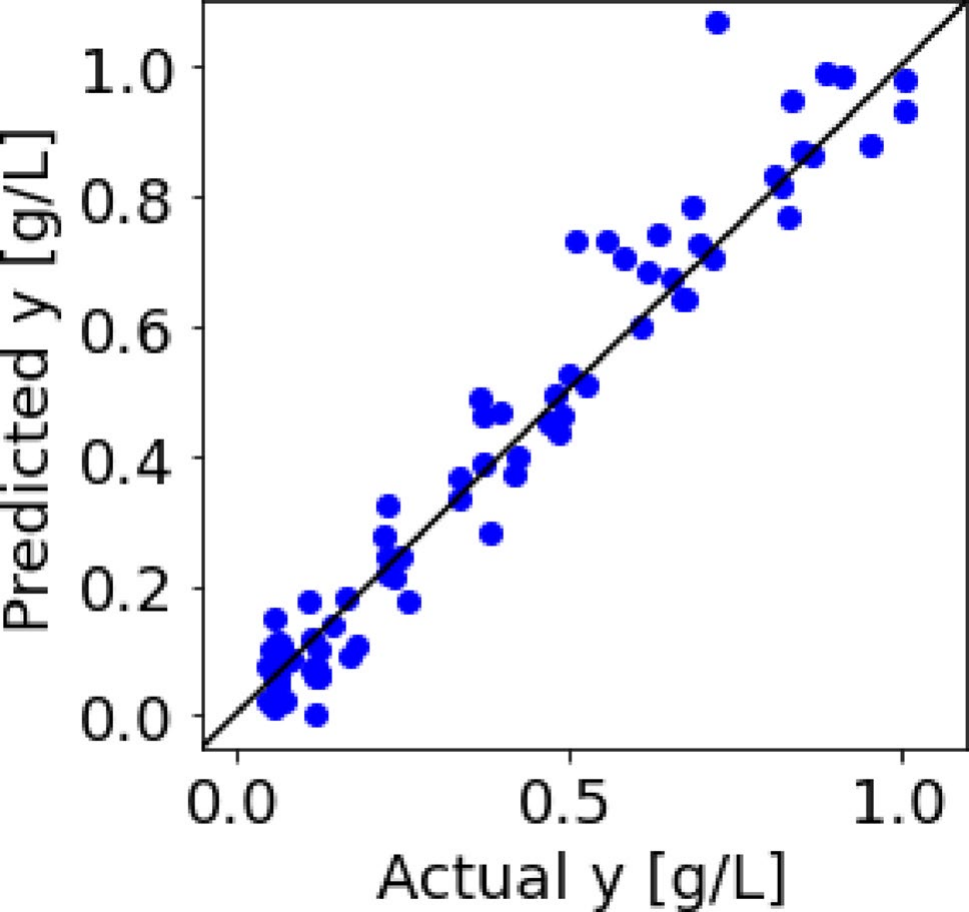
Actual y versus predicted y in test data of the batch process data set

**Fig. 9:**
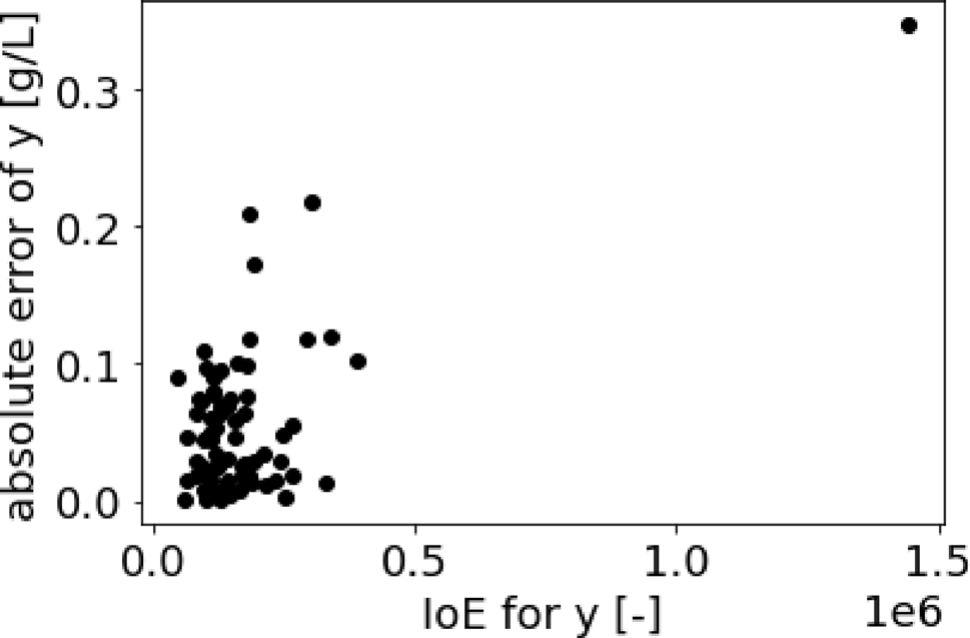
IoE for y versus absolute error of y in test data of the batch process data set

The results of building the GMR model using the training data and predicting the y values for the test data are shown in Fig. 8, and the distribution of IoE and absolute prediction errors of y in the test data is shown in Fig. 9. The sample with a large prediction error had a large IoE value, indicating that IoE can flag samples located in low-density regions of the training data. Because high IoE corresponds to lower prediction reliability, the variance or dispersion of prediction errors is expected to be larger in such regions. These results show that IoE is useful for identifying predictions that should be interpreted with caution in forward analysis.

**Fig. 10:**
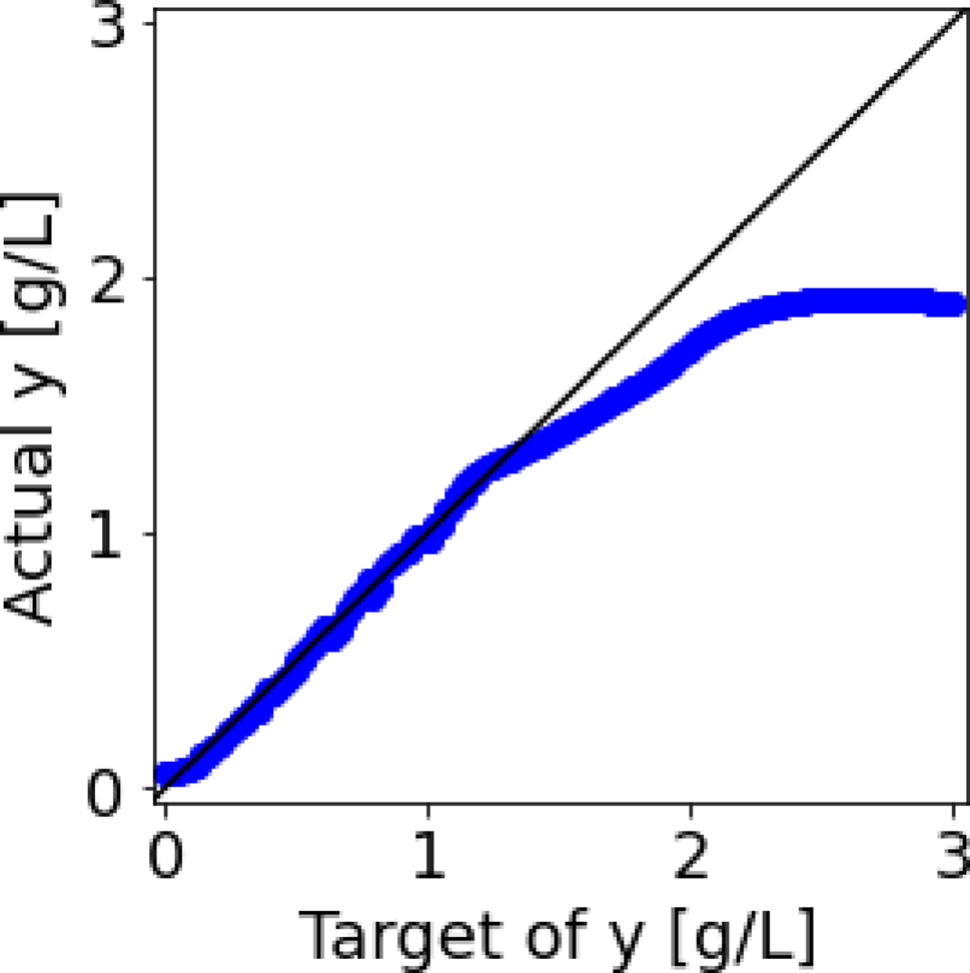
Target y versus actual y as the results of the DIA of the GMR model

**Fig. 11:**
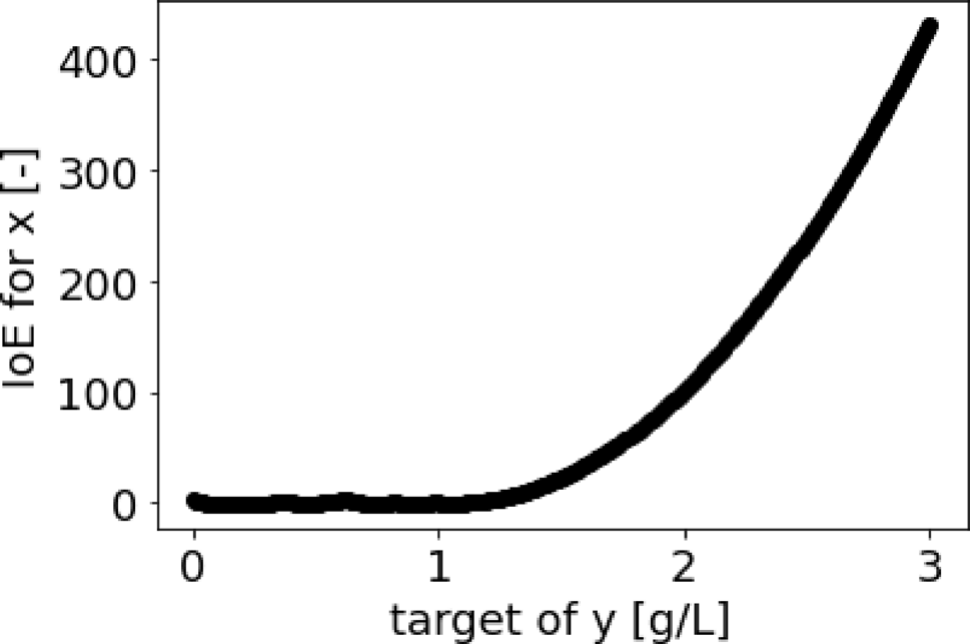
Target of y versus IoE for y as the results of the DIA of the GMR model

**Fig. 12:**
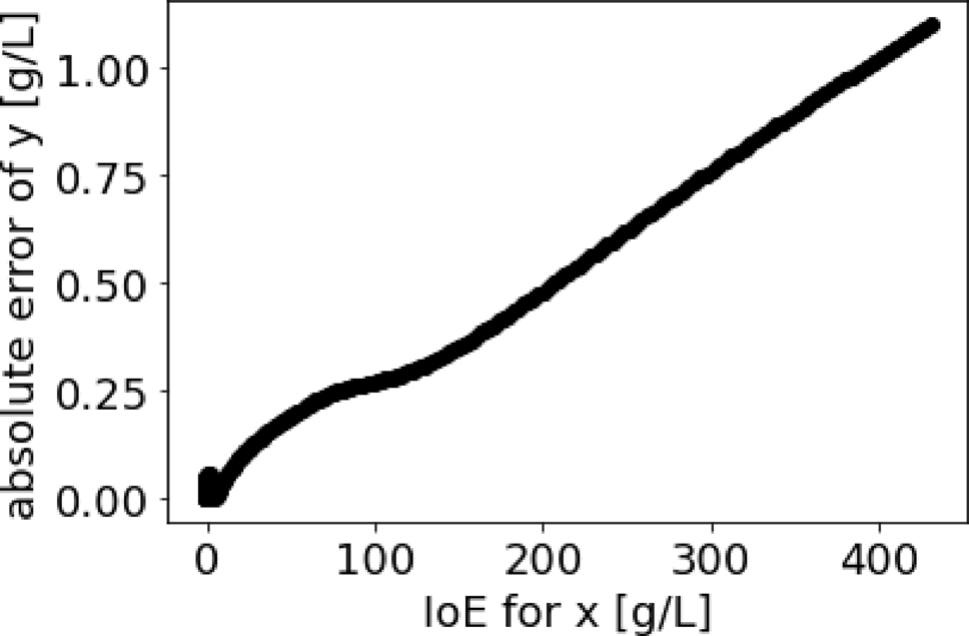
IoE for x versus absolute error of y as the results of the DIA of the GMR model

The target y value was varied from 0 to 3 in 0.01 increments. For each target y value, x values were directly predicted by DIA of the GMR model, and the actual y value was obtained by simulating the process using Eqs. (5)–(13) based on the predicted x values. A plot between the target y value and the actual y value is shown in Fig. 10. The maximum value of y in the 300 batches for model building was 1.26; therefore, target values of y above 1.26 were considered extrapolative in the y space. The difference between the target y value and the actual y value gradually increased when the target y value exceeded 1.26. A plot of the target y value versus IoE is shown in Fig. 11, and a plot between IoE and the absolute difference between the target y value and the actual y value is shown in Fig. 12. As the target y value increased beyond the training data domain, IoE also increased, indicating reduced prediction reliability in the extrapolative y region. In this context, reduced reliability means a larger dispersion of the prediction-error distribution, and therefore large errors may occur more readily; however, a high IoE does not imply that every realized error must be large.

From the above results, IoE was confirmed to evaluate whether the input information is located in an extrapolative region in both forward and inverse analyses of the GMR model. IoE should be interpreted as a qualitative reliability indicator that flags low-density regions where the prediction-error distribution may have larger variance or dispersion, rather than as a deterministic estimator of the actual error.

## Conclusions

In this study, an index of extrapolation (IoE) was proposed to evaluate the reliability of prediction results in both forward and inverse analyses of Gaussian mixture regression (GMR) models. Although GMR and direct inverse analysis (DIA) can effectively predict relationships between features and target variables, the lack of a quantitative measure to assess whether predictions lie within interpolation or extrapolation regions has limited their reliability. The proposed IoE, defined as the negative logarithm of the probability density function (PDF), enables the identification of extrapolated samples in a mathematically stable manner.

Through numerical simulations, it was confirmed that IoE successfully distinguished interpolation-like and extrapolation-like regions, where larger IoE values corresponded to lower probability density and greater extrapolation potential. Furthermore, applications to data sets of organic compounds, inorganic materials, and a batch process demonstrated that high-IoE regions correspond to reduced prediction reliability, in which the prediction-error distribution may have larger dispersion. These results indicate that IoE can serve as a practical diagnostic indicator of prediction reliability. Because IoE is based on probability density, it should be interpreted as a qualitative warning index rather than a deterministic predictor of the exact prediction error.

The significance of this study lies in bridging a critical gap between model prediction and its practical applicability. The IoE not only identifies the limits of reliable prediction but also enables practitioners to evaluate, in advance, whether target conditions fall inside or outside the applicability domain. This capability is particularly valuable in molecular, material, and process design, where extrapolative predictions often guide the discovery of novel systems.

Overall, the proposed IoE provides a versatile and generalizable framework that strengthens the methodological foundation of data-driven design. Beyond enhancing interpretability, it lays the groundwork for integration with active learning and adaptive experimental strategies, accelerating the deployment of machine learning in chemical engineering and materials informatics.

## Data Availability

The data that support the findings of this study are available from the corresponding author upon reasonable request.
